# Space physiology: Challenges and solutions for a journey to Mars

**DOI:** 10.1113/EP093375

**Published:** 2026-08-27

**Authors:** Damian M. Bailey

**Affiliations:** ^1^ Neurovascular Research Laboratory, Faculty of Life Sciences and Education University of South Wales Pontypridd UK; ^2^ Bexorg New Haven Connecticut USA

## 1 TO THE MOON AND MARS: PHYSIOLOGY BEYOND LOW EARTH ORBIT

The central biomedical challenge for human exploration is not simply how far spacecraft can travel but whether human physiology can adapt safely to prolonged life beyond Earth. Spaceflight provides a unique testbed for defining the limits of physiological adaptation and resilience. The human body, adapted to Earth's gravitational environment, must respond simultaneously to microgravity or partial gravity, ionising radiation, altered atmospheric composition, circadian disruption and confinement. Determining when these responses progress from adaptation to deconditioning, disease and impaired operational performance is essential for crew health and mission success. The resulting insights also inform ageing, physical inactivity, critical illness, musculoskeletal and cardiovascular disease, and healthcare delivery in remote or resource‐limited environments.

This is a particularly timely period for space physiology. On 6 April 2026, Artemis II conducted the first crewed lunar flyby in >50 years, with subsequent Artemis missions intended to support sustained human activity on and around the Moon and prepare for future missions to Mars (National Aeronautics & Space Administration, 2026). Beyond low Earth orbit, longer mission durations, delayed communications, the absence of rapid evacuation, and sustained exposure to interacting environmental stressors fundamentally alter the biomedical context. Crew members will consequently require greater autonomy in monitoring and managing their health.

Together, these environmental and operational hazards comprise the space exposome, i.e., the cumulative mission exposures and their interactions with individual susceptibility. The space integrome describes the corresponding integrated, multiscale biological response across genes, molecules, cells, tissues, organs, physiological systems and behaviour, which collectively determines adaptation, resilience or maladaptation (Bailey, 2025; Bailey et al., 2025). Deeper longitudinal phenotyping of the exposome–integrome interaction is therefore needed to determine how combined stressors modify individual risk and to guide personalised countermeasure selection. Although >30 health risks have been associated with exploration‐class missions, the additive, antagonistic and non‐linear effects of combined exposures remain poorly characterised (Bailey, 2025; Bailey et al., 2025).

These uncertainties create a clear role for physiologists in translating mechanistic insight into practical intervention. Exercise, artificial gravity, nutrition, pharmacology, environmental control and digital health monitoring must be evaluated both individually and as coordinated, mission‐specific countermeasure packages tailored to the astronaut and operational context. Building this capability will require sustained investment in doctoral training, fellowships and interdisciplinary career pathways spanning physiology, aerospace medicine, engineering and data science.

The need for integrated evidence and constructive scientific exchange motivated the *Experimental Physiology* Special Issue, ‘Space physiology: challenges and solutions for a journey to Mars’. Its contributions span original research articles, reviews, editorials, viewpoints, case reports, short communications, letters, Myths and Methodologies articles and Methods and Techniques reports, reflecting broad engagement across the field.

This editorial synthesises the collection across five interconnected themes: integrated and personalised risk; cerebral, neuro‐ocular and sensorimotor adaptation; exercise countermeasures and operational performance; tissue‐specific responses to altered gravitational loading; and the translation of physiological evidence into operational medicine, training and scientific discourse. Together, these themes move from characterising the hazards of exploration‐class spaceflight towards defining the measurements, experimental models and countermeasures required to mitigate them.

## 2 FROM EXPOSURE TO ADAPTATION: WHAT THIS SPECIAL ISSUE TEACHES US

### 2.1 Integrated and personalised risk

A central principle emerging from the collection is that astronaut health cannot be understood by examining individual organs or environmental stressors in isolation. The Viewpoint ‘Decoding the space integrome’ advocates longitudinal profiling and countermeasures tailored to the astronaut, mission profile and cumulative exposome burden (Bailey, 2025; Bailey, et al., 2025). This framework encompasses space omics, where next‐generation sequencing and mass spectrometry enable high‐throughput genomic, transcriptomic, proteomic and metabolomic profiling from small biological samples. Minimally invasive sampling, including dried blood spots, capillary blood, saliva and urine, further strengthens the operational potential of these approaches. However, translation of molecular signatures into validated biomarkers remains constrained by small cohorts, interindividual variability and insufficient methodological standardisation.

Radiation exemplifies a major risk that remains difficult to reproduce experimentally and to mitigate operationally. Edwards et al. (2024) demonstrate that low‐dose X‐ray preconditioning confers modest protection against subsequent exposure to high‐dose X‐ray or simulated galactic cosmic radiation in cultured cells. Although not proposed as a practical countermeasure, these findings identify inducible cellular defence pathways that might provide mechanistic targets for safer radioprotective strategies.

Personalised risk assessment must also account for sex and life stage. Hughes‐Fulford et al. (2024) identify persistent gaps in female‐specific evidence relating to orthostatic tolerance, musculoskeletal loss, immunity, urinary health, radiation risk and spaceflight‐associated neuro‐ocular syndrome (SANS). Holden (2025) considers reproduction during interplanetary flight and highlights potential risks to implantation, fetal development and subsequent neurological health. Kern et al. (2026) frame spaceflight as an accelerated model of biological stress, through which to investigate ageing, renal dysfunction and cellular senescence, while considering dynamically targeted pharmacological countermeasures. Collectively, these contributions emphasise that increasingly diverse crews require an equally representative and life‐stage‐specific evidence base.

Ground‐based analogues remain essential for developing that evidence. Hajj‐Boutros et al. (2024) describe the Canadian Bedrest Study as a platform for examining inactivity‐induced multisystem decline, while Fernandez‐Gonzalo et al. (2024) define the strengths and limitations of head‐down‐tilt bed rest as an analogue of microgravity‐induced deconditioning. Because no single model reproduces the complete space exposome, each analogue must be judged according to the exposure or physiological process it reproduces, the fidelity achieved and the question it is designed to address. Together, these papers move risk assessment beyond isolated hazards towards the integrated evaluation of interacting exposures, individual susceptibility and early biomarkers of physiologically or operationally meaningful change.

### 2.2 The cerebral–ocular–vascular axis: Integrated responses to altered gravity

The cerebral–ocular–vascular axis is particularly sensitive to cephalad fluid redistribution, altered atmospheric composition, vascular remodelling and disturbed sensory integration. Carr et al. (2025) review the cerebral consequences of chronic exposure to elevated inspired CO_2_ in confined habitats. Sustained hypercapnia and respiratory acidosis can alter cerebral blood flow, intracellular proton concentration, metabolism, cognition and visuomotor performance, particularly when combined with fluid redistribution, sleep disruption and operational workload. Supporting multistressor study designs, Kato et al. (2024) demonstrate that graded head‐down tilt combined with 3% inspired CO_2_ impairs dynamic cerebral autoregulation at steeper tilt angles.

Interpretation of spaceflight‐related changes in cerebral blood flow also depends crucially on the reference condition. Possnig et al. (2025) report reductions in global and regional cerebral blood flow during 3 days of bed rest, although values did not fall below those measured in the upright terrestrial posture. Following 6 months of spaceflight, Fournier et al. (2025) found that middle cerebral artery pulsatility increased in some astronauts by an amount comparable to 5–10 years of terrestrial ageing. These individual changes were associated with increased pulse pressure and carotid artery stiffness, linking central vascular remodelling to greater pulsatile load within the cerebral circulation.

Neurovestibular adaptation presents an immediate operational risk. Kravets & Clark (2024) report that a ∼30% overestimation of head tilt persisted for 1 h of controlled hypergravity exposure. Their experimentally informed computational model might help to predict sensory misperception during transitions between gravitational environments, when crews may be required to land, egress or respond rapidly to emergencies.

SANS remains a complex and incompletely understood consequence of long‐duration spaceflight. It is characterised by variable combinations of reduced near visual acuity, hyperopic refractive shifts, optic disc oedema, posterior globe flattening, chorioretinal folds, cotton‐wool spots and altered retinal nerve‐fibre‐layer thickness, often presenting asymmetrically. Ng and Mollan (2025) review these ocular changes, the continuing uncertainty surrounding intracranial pressure and one‐carbon metabolism, and emerging countermeasure options.

Tang et al. (2026) provide longitudinal imaging evidence that cerebral and ocular changes following long‐duration spaceflight reflect a shared cranial response to cephalad fluid redistribution rather than an isolated ocular disorder. Third‐ventricle expansion was associated selectively with changes in globe geometry, but not with changes in the optic nerve, optic nerve sheath or other retro‐orbital structures. These structure‐specific relationships support disrupted and compartmentalised CSF dynamics as a potential mechanism of SANS.

The accompanying Viewpoints extend this interpretation. Zu Eulenburg et al. (2025) propose the cranial microgravity response as a unifying framework for the interconnected cerebral, ocular and CSF adaptations to microgravity. Mollan and Bailey (2026) subsequently argue that CSF compartmentalisation offers a more physiologically integrated explanation than a uniform increase in intracranial pressure intracranial pressure. This distinction is operationally important, because countermeasures might need to target regional CSF flow, fluid redistribution and tissue biomechanics rather than simply reducing global pressure alone.

Faivre‐Rampant et al. (2026) reinforce the importance of physiologically appropriate analogue selection. Head‐up tilt and lower‐body negative pressure produced different regional fluid shifts and haemodynamic responses despite matched reductions in thoracic blood volume. Equivalence in a single variable therefore does not imply equivalence of the integrated physiological response.

Collectively, these studies support an integrated cerebral–ocular–vascular framework linking fluid redistribution, vascular function, CSF dynamics, tissue biomechanics and sensory perception. More broadly, they emphasise that effective countermeasures must address interacting physiological systems within mission‐specific operational constraints.

### 2.3 Exercise countermeasures: From physiological prescription to operational delivery

Exercise remains the principal systemic countermeasure in human spaceflight, but deep‐space missions will impose greater constraints on equipment mass, volume, power, maintenance and crew time. The central challenge is therefore how to deliver a sufficient multisystem stimulus reliably within these limits.

Fernandez‐Gonzalo et al. (2025) broaden the definition of an effective exercise countermeasure beyond mechanical loading to include interindividual responsiveness, periodisation, nutrition, adherence, enjoyment and multi‐organ benefit. DeVirgiliis et al. (2025) extend this framework through the astronaut–athlete paradigm, applying principles from sport science to preflight preparation, longitudinal monitoring, exercise prescription, recovery, sleep and nutrition.

At the tissue level, Bosutti et al. (2025) review why current programmes do not fully preserve skeletal muscle mass and function, highlighting the difficulty of reproducing Earth‐like loading and the potential role of neuromuscular electrical stimulation. Matsakas and Deane (2025) place these mechanisms within a broader translational framework, while McMahon et al. (2025) propose high‐intensity isometric resistance training at longer muscle–tendon complex lengths as a compact, mechanically efficient strategy to preserve neuromuscular function and mass during microgravity exposure.

Blood flow restriction (BFR) is examined from physiological prescription to operational implementation. Swain et al. (2024) demonstrate that lower‐limb occlusion pressure varies with body inclination and the unloading model, indicating that pressure should be determined in the same posture and environment in which BFR will be applied. Laflamme et al. (2025) subsequently show that personalised BFR exercise is feasible during parabolic‐flight microgravity, augmenting cardiovascular and perceptual demands while remaining tolerable. Fox et al. (2026) extend the discussion to the neurophysiological mechanisms and broader translational potential of BFR.

Swain et al. (2025) examine bodyweight jumping in simulated lunar gravity and report graded increases in oxygen consumption, heart rate, ventilation, blood lactate and ground‐reaction force with jump height. These findings support jumping as a low‐hardware strategy for concurrent cardiovascular and musculoskeletal loading, although its longer‐term skeletal effects, safety and operational acceptability require further evaluation.

Hardware development is represented by the ATHLETIC exoskeleton and NEX4EX multipurpose exercise device. ATHLETIC enabled resistive and plyometric exercise but delivered lower loading and muscle activation than reference movements (Böcker, Zange, Gruber et al., 2025). NEX4EX combined resistive, plyometric and sensorimotor exercise and elicited appropriate postural reflexes, although exercise loads and jump velocities were again lower than terrestrial comparators (Böcker, Zange, Siedel et al., 2025). Together, these studies identify the engineering and physiological refinements required for compact devices to deliver an adequate training stimulus before deployment.

The accompanying Viewpoint by De Martino et al. (2025) considers how compact, multimodal hardware might satisfy exploration constraints, while Watenpaugh & Hargens (2026) argue that lower‐body negative‐pressure exercise should remain under consideration. This exchange highlights the need to compare competing countermeasure strategies against standardised physiological, operational and behavioural outcomes.

Collectively, these contributions support multimodal exercise systems combining efficient mechanical loading with individualised prescription, longitudinal monitoring and adjunct strategies such as neuromuscular electrical stimulation or BFR. Their effectiveness will depend less on the selected modality than on the magnitude, distribution and tissue specificity of the physiological stimulus delivered.

### 2.4 Gravity, loading and the musculoskeletal axis

The consequences of unloading extend beyond muscle. Bone, intervertebral discs, connective tissues and sensorimotor control adapt over different time scales and may respond differently to a given gravitational or exercise stimulus. Liu et al. (2025) report that 60 days of head‐down‐tilt bed rest caused foot pain and reduced calcaneal bone mineral density, neither of which was prevented by continuous or intermittent artificial gravity. Bone marrow adipose tissue was unchanged, while exploratory analyses suggested sex‐specific differences in bone loss. Together, these findings show that restoring a gravitational vector does not necessarily provide the tissue‐specific magnitude, distribution or duration of loading required for protection.

The complementary lumbar and cervical studies by Marcos‐Lorenzo et al. (2024, 2026) demonstrate rapid, region‐specific spinal responses to unloading. Four hours of hyper‐buoyancy flotation increased stature and lumbar intervertebral disc height and induced moderate back pain; 30 s of seated axial loading at 50% bodyweight attenuated these changes to a similar extent to 15 min of upright sitting, without altering passive vertebral stiffness. In the cervical spine, the same unloading stimulus increased disc height, reduced passive vertebral stiffness and caused mild neck pain, with only partial recovery after 15 min of upright loading. The differing associations with pain (lumbar pain with disc expansion and stature gain, and cervical pain with reduced vertebral stiffness) suggest distinct regional mechanisms and support further evaluation of brief, targeted axial‐loading countermeasures.

Reliable analogue systems are essential for characterising these responses and testing countermeasures. Swain et al. (2026) introduce the Variable Gravity Suspension System as an accessible platform for simulating microgravity and partial‐gravity locomotion and jumping. Alongside bed rest, flotation and cardiovascular unloading models, this work reinforces a central methodological principle that analogue validity should be judged against the physiological process under study, rather than against a general claim to reproduce spaceflight.

Gravitational countermeasures must therefore be prescribed according to the tissue‐specific dose, direction, duration and timing of loading. Restoration of some loading is not equivalent to restoration of the biological stimulus normally provided by 1 *g*. Their operational value will depend on delivering that stimulus safely, consistently and within exploration‐class mission constraints.

### 2.5 From physiological evidence to operational implementation

The final theme concerns the translation of physiological evidence into mission operations. Fall and Bailey (2026) draw attention to coagulation and thrombotic risk, proposing a model in which upstream free‐radical‐mediated procoagulant signalling, endothelial activation and altered haemodynamics interact to increase thrombotic risk. Puhl and Magkos (2025) consider how commercial low‐Earth‐orbit missions might broaden access to participants, research platforms and biological samples, helping to address limitations in cohort size and demographic representation, provided that methods, metadata and data access are harmonised across operators.

Several editorials broaden the scientific and translational perspective of the collection. Bailey and van Ombergen (2025) examine the limits of human adaptation and the relevance of space physiology to health on Earth, while Kern and Siew (2026) frame space biology as a catalyst for biotechnological and longevity‐focused innovation, and Hartmann et al. (2025) demonstrate how popular culture through *The Adventures of Tintin* can provide an accessible introduction to the physiological challenges of spaceflight.

Training must also progress alongside the science. Deane (2024) argues for meaningful access, visibility and responsibility for early career researchers. Recruitment and retention will depend not only on communicating the appeal of the field, but also on establishing funded training and career pathways that convert interest into sustained expertise.

The accepted collection includes Editorials, Viewpoints and a Letter, all centred on interpretation, challenge and debate. This is especially valuable in a field constrained by small cohorts, heterogeneous missions and imperfect analogues. Exchanges on exercise‐countermeasure design and SANS demonstrate how constructive disagreement can separate evidence from extrapolation and define the studies needed to resolve uncertainty. Sustained exploration will therefore require operationally relevant biomedicine, interdisciplinary training, coordinated research platforms and transparent scientific scrutiny, priorities that align directly with the wider community‐led strategy outlined below.

## 3 FROM EVIDENCE GAPS TO A SHARED RESEARCH STRATEGY

The priorities emerging from this Special Issue align closely with the European Space Agency's SciSpacE White Papers, which connect community‐led fundamental science to the applied research needed to support the Terrae Novae exploration programme (European Space Agency, 2021). The collection advances several of these priorities, including integrated risk assessment, sex‐specific physiology, radiation biology, cerebrovascular and neuro‐ocular adaptation, musculoskeletal deconditioning, analogue validation, precision monitoring and countermeasure development.

**FIGURE 1:**
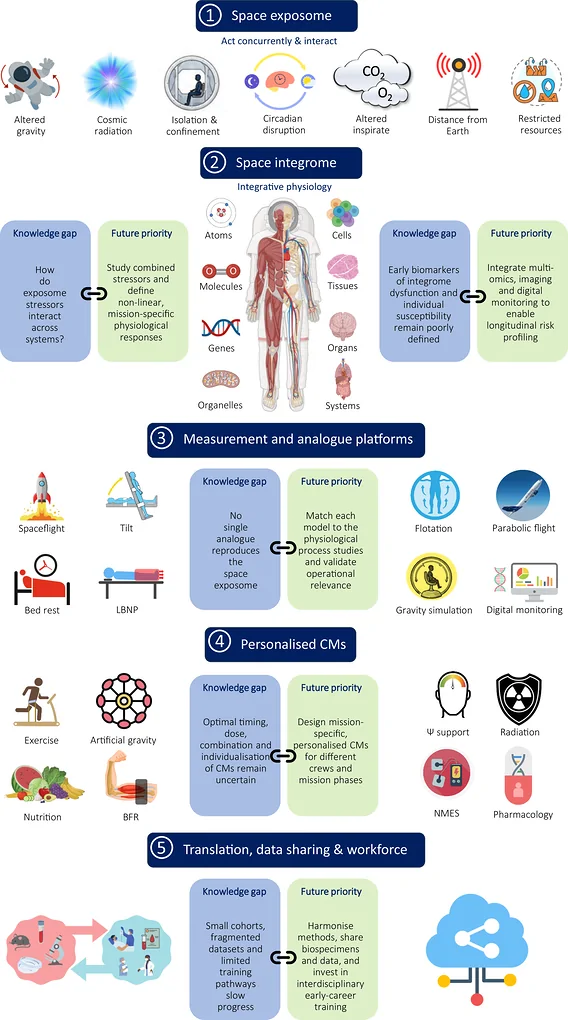
Knowledge gaps and future priorities in space physiology. Schematic diagram illustrating the progression from interacting space–exposome stressors to the integrated, multiscale space integrome and the principal knowledge gaps identified across this Special Issue. Priorities include characterising combined stressor responses, validating early biomarkers of individual susceptibility, improving the physiological fidelity of spaceflight analogues and determining the optimal dose, timing, combination and personalisation of countermeasures. Progress will also require harmonised methods, shared biospecimens and datasets and sustained investment in interdisciplinary training and workforce development. Abbreviations: ψ, Psychological; BFR, blood flow restriction; CMs, countermeasures; LBNP, lower‐body negative pressure; NMES, neuromuscular electrical stimulation. Figure created in BioRender.

However, important gaps remain. Interacting components of the space exposome remain difficult to reproduce experimentally; early biomarkers of integrome dysfunction remain insufficiently validated; and the optimal dose, timing and combination of many countermeasures remain uncertain. Addressing these gaps will require harmonised methods, longitudinal sampling and wider sharing of data and biospecimens among agencies, commercial operators and research groups. Analogue studies must establish their physiological and operational relevance, and mission studies should generate mechanistic insight that extends beyond individual flights. Sustained funding, fellowships and interdisciplinary career pathways are equally essential to retain expertise between missions and equip early career researchers to develop the countermeasures needed for sustained lunar operations and future Mars exploration. These interconnected gaps and future priorities are summarised in Figure 1.

This Special Issue does not resolve every biomedical challenge of deep‐space flight, but it defines a clearer route forwards by linking deep longitudinal phenotyping with validated analogues, mission‐ready countermeasures and shared data standards. Engineering will take us beyond low Earth orbit; integrative physiology will determine whether crews can remain healthy and operationally effective once there. My sincere thanks to all the authors, reviewers and Journal and Society staff who made this collection possible! I hope it stimulates collaboration, constructive debate and the next generation of research needed to carry human physiology safely to the Moon, Mars and beyond.

## AUTHOR CONTRIBUTIONS

Damian M. Bailey conceived the idea and wrote the first draft of the manuscript. He approved the final version submitted for publication and agrees to be accountable for all aspects of the work in ensuring that questions related to the accuracy or integrity of any part of the work are appropriately investigated and resolved. All persons designated as authors qualify for authorship, and all those who qualify for authorship are listed.

## CONFLICT OF INTEREST

Damian M. Bailey is Editor‐in‐Chief of *Experimental Physiology* and outgoing Chair of the Life Sciences Working Group and outgoing member of the Human Spaceflight and Exploration Science Advisory Committee to ESA. Damian M. Bailey is a current member of the ESA‐HRE‐Biology Panel and Space Exploration Advisory Committees to the UK and Swedish National Space Agencies.

## GENERATIVE AI STATEMENT

No generative AI tools were used in the preparation of this manuscript.
